# Pharmacological cGAS-STING pathway inhibition on neuroinflammation in animal models of traumatic brain injury: A systematic review and meta-analysis protocol

**DOI:** 10.1371/journal.pone.0362397

**Published:** 2026-11-06

**Authors:** Myriam Lamamri, Stéphanie Sigaut, Hélène Cwerman-Thibault, Valérie Faivre, Juliette Van Steenwinckel, Vincent Degos, Pierre Gressens, Alice Jacquens

**Affiliations:** 1 Département d’anesthésie réanimation, AP-HP, Hôpital Beaujon, DMU PARABOL, Clichy, France; 2 Université Paris Cité, Inserm, NeuroDiderot, Paris, France; 3 Département d’anesthésie réanimation, AP-HP, Hôpital La Pitié Salpétrière, DMU DREAM, Paris, France; Neyshabur University of Medical Sciences, IRAN, ISLAMIC REPUBLIC OF

## Abstract

**Background:**

Traumatic brain injury (TBI) is a major cause of mortality and long-term neurological disability worldwide. Secondary injury mechanisms, particularly neuroinflammation, play a key role in determining neurological outcomes after the initial trauma. The cyclic GMP-AMP synthase stimulator of interferon genes (cGAS-STING) pathway has recently been identified as a mediator of innate immune activation following brain injury. Activation of this pathway promotes microglial activation and the release of pro-inflammatory cytokines, contributing to neuronal damage and impaired recovery. Pharmacological inhibition of the cGAS-STING signaling pathway has therefore emerged as a promising neuroprotective strategy in experimental TBI models. However, the available preclinical evidence has not yet been systematically synthesized.

**Methods:**

A systematic literature search was performed across MEDLINE, EMBASE and Web of Science without language or publication date restrictions. Titles and abstracts were screened independently by two reviewers, followed by independent full-text eligibility assessment. Data extraction was initiated subsequently and will be completed independently by two reviewers. Eligible studies will include in vivo animal models of TBI investigating direct or indirect pharmacological inhibition of the cGAS-STING pathway administered after injury, compared with vehicle-treated or untreated controls. The primary outcome will be quantitative measures of microglial activation within the first 72 hours post-injury, preferentially assessed using Iba1-positive cell density or percentage-stained area. Secondary outcomes will include astrocytic reactivity, cytokine expression (TNF-α, IL-1β, IL-6), lesion volume and neurological functional recovery. Risk of bias will be assessed using the SYRCLE tool. When appropriate, pooled effect sizes will be calculated using random-effects meta-analysis. Heterogeneity, publication bias and certainty of evidence will also be evaluated.

**Discussion:**

This systematic review and meta-analysis will help clarify the magnitude and consistency of therapeutic effects and inform the translational potential of this strategy while identifying methodological limitations that may guide future experimental and clinical research.

**Systematic review registration:**

PROSPERO CRD420261367166.

## Introduction

Traumatic brain injury (TBI) is a leading cause of death and long-term disability worldwide, particularly among young adults [1]. Beyond the immediate primary injury, the neurological outcome following TBI is largely determined by secondary injury mechanisms that evolve over hours to weeks after the initial trauma [2]. Among these processes, neuroinflammation plays a central and sustained role, contributing to neuronal loss, synaptic dysfunction and chronic neurodegeneration [3,4]. Despite extensive research efforts, no pharmacological intervention targeting post-traumatic neuroinflammation has yet demonstrated clear clinical efficacy, highlighting a persistent gap in the clinical management of TBI patients [5].

Preclinical animal models of TBI have been essential in elucidating the molecular and cellular mechanisms underlying secondary brain injury [6,7]. These models consistently demonstrate early activation of innate immune responses, characterized by microglial activation, cytokine release and leukocyte infiltration [8–10]. While neuroinflammation is initially protective, its dysregulation and persistence are associated with worsened neurological recovery [11]. As a result, targeting key upstream regulators of innate immune activation has emerged as a promising strategy to mitigate secondary injury and improve long-term outcomes [12,13].

In recent years, the cyclic GMP-AMP synthase stimulator of interferon genes (cGAS-STING) pathway has been identified as a pivotal driver of neuroinflammatory responses following TBI [14]. Originally characterized as a cytosolic DNA-sensing pathway involved in antiviral immunity, the cGAS-STING axis is now recognized as a sensor of endogenous danger-associated molecular patterns released after traumatic injury [15,16]. Experimental studies have shown that mitochondrial and nuclear DNA released into the cytosol activates cGAS, leading to STING-dependent signaling, activation of interferon regulatory factor 3 (IRF3) and nuclear factor kappa B (NF-κB), and sustained production of type I interferons and pro-inflammatory cytokines [17]. This pathway has been implicated in microglial activation and neuronal damage across multiple preclinical models of TBI [14,15,18,19].

Pharmacological inhibition of the cGAS-STING pathway has therefore gained increasing attention as a potential neuroprotective strategy. Small-molecule inhibitors targeting STING have been evaluated in animal models of TBI, with reported effects including reduced microglial activation, attenuation of pro-inflammatory cytokine expression, decreased lesion volume and improved functional outcomes [18,20,21]. However, these studies vary widely in terms of animal species, injury models, timing and duration of treatment, outcome measures, and methodological quality. Moreover, concerns regarding risk of bias, selective reporting and limited reproducibility remain pervasive in preclinical neurotrauma research.

At the time of PROSPERO registration on 12 April 2026, we checked the registry and the main bibliographic databases, including PubMed, Embase and Web of Science, for existing or ongoing systematic reviews and meta-analyses on direct or indirect pharmacological inhibition of the cGAS-STING pathway in preclinical models of TBI. No published systematic review, meta-analysis, or registered review protocol addressing this specific question was identified. Without such synthesis, it remains unclear whether the observed effects are consistent, robust and biologically meaningful, or whether they are driven by isolated findings or methodological limitations. A meta-analysis is therefore essential to quantify the magnitude of therapeutic effects, assess between-study heterogeneity, evaluate risk of bias, and appraise the overall certainty of the evidence [22].

By systematically evaluating the efficacy of pharmacological cGAS-STING pathway inhibition on neuroinflammation and related outcomes in animal models of TBI, this review aims to clarify the translational potential of this therapeutic strategy. Such synthesis is important to inform future experimental design and to support rational progression toward clinical investigation. Despite promising preclinical findings, numerous anti-inflammatory strategies have failed in clinical TBI trials, highlighting the need for preclinical evidence synthesis before clinical translation [5,23].

Our primary objective will be to systematically evaluate the efficacy of the direct or indirect pharmacological inhibition of the cGAS-STING pathway on microglial activation following TBI in animal models. Our secondary objectives will be:

- To evaluate the impact of direct or indirect pharmacological cGAS-STING inhibition on astrocytic reactivity assessed by glial fibrillary acidic protein (GFAP) expression
- To evaluate the impact of direct or indirect pharmacological cGAS-STING inhibition on neurological functional recovery (motor and cognitive outcomes)
- To evaluate the impact of direct or indirect pharmacological cGAS-STING inhibition on lesion volume
- To evaluate the impact of direct or indirect pharmacological cGAS-STING inhibition on the expression (messenger ribonucleic acid or mRNA and protein levels) of pro-inflammatory cytokines: tumor necrosis factor alpha (TNF-α), IL − 1β and IL-6.

## Methods

### Study design

This protocol describes an ongoing systematic review and planned meta-analysis of published preclinical studies involving animal models of TBI and evaluating direct or indirect pharmacological inhibition of the cGAS-STING pathway. The review question was formulated according to the PICO framework (Table 1). The protocol followed the preferred reporting items for systematic reviews and meta-analysis protocols (PRISMA-P) guidance (S1 File) [24]. We also followed the guidance from the Systematic Review Centre for Laboratory Animal Experimentation (SYRCLE) for preparation, registration and publication of systematic reviews of animal intervention studies (S2 File) [25]. The protocol was registered in PROSPERO (CRD420261367166). A PRISMA flow diagram will be produced in the completed systematic review and meta-analysis to report the number of records identified, screened, included and excluded, together with the reasons for full-text exclusions (Fig 1). This systematic review will include both qualitative and quantitative syntheses. This study is a protocol for a systematic review and meta-analysis of published preclinical studies and does not involve new studies with human participants, human data or animals.

**Table 1:** PICO framework for the review question.

PICO element	Description
**Population**	In vivo animal models of TBI, regardless of species, sexes, ages or injury severity.
**Intervention**	Direct or indirect pharmacological inhibition of the cGAS-STING pathway administered after TBI induction.
**Comparator**	Vehicle-treated or untreated TBI animals.
**Primary outcome**	Quantitative microglial activation assessed within the first 72 hours post-injury.
**Secondary outcomes**	Astrocytic reactivity assessed by glial fibrillary acidic protein (GFAP); pro-inflammatory cytokine expression (mRNA and protein levels of TNF-α, IL-1β, and IL-6); lesion volume and neurological functional recovery.

**Fig 1:**
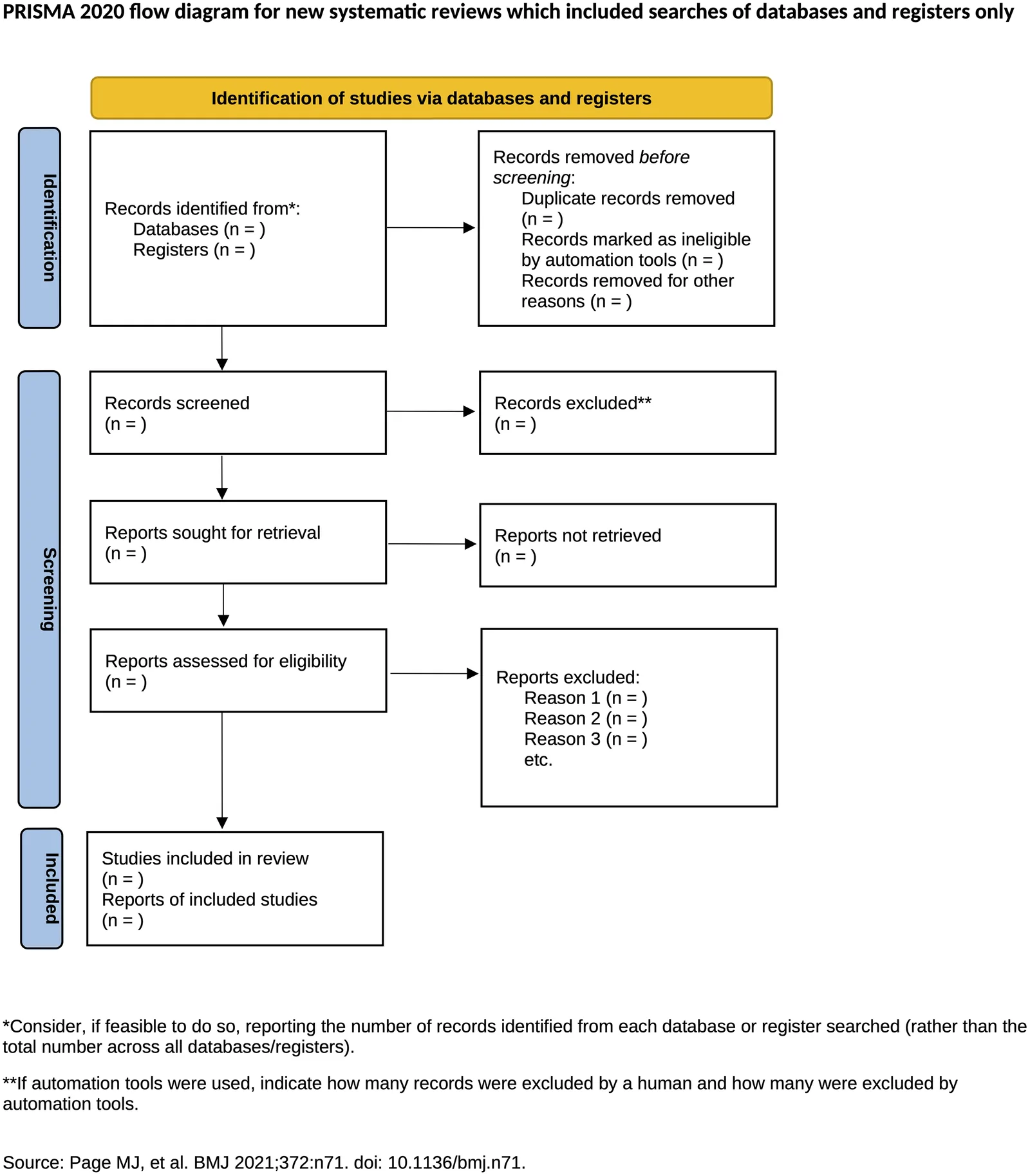
PRISMA flow diagram.

### Status and timeline of the study

At the time of PROSPERO registration on 12 April 2026, only preliminary scoping searches and development of the search strategy had been undertaken. After protocol registration, the database search and study selection process was performed from inception to September 2026. In parallel, data extraction was initiated from July 2026 onwards as studies were confirmed eligible for inclusion. The most recent search of the bibliographic databases to identify eligible studies was performed on 1 September 2026. The results of the systematic review and meta-analysis are expected by January 2027.

### Search strategy

We systematically searched MEDLINE (via PubMed), Embase (Elsevier) and Web of Science from inception, without language or publication date restrictions. Only records indexed in bibliographic databases and published as full-text articles in peer-reviewed journals were considered. Grey literature sources, including conference abstracts, dissertations, theses, editorials, and non-peer-reviewed reports, were not searched because these sources frequently provide insufficient methodological and quantitative detail for robust risk-of-bias assessment and effect-size estimation. However, to reduce the risk of missing relevant evidence and to better assess potential publication bias, an updated sensitivity search will be performed after completion of the data extraction and before the analyses. As part of this final search process, the reference lists of all included articles and relevant reviews and preprint servers will be screened. Any additional potentially eligible records identified through these sources will be assessed using the same eligibility criteria as database records.

The electronic search strategies were developed with the assistance of a medical librarian and adapted to the controlled vocabulary and syntax of each database. They combined database-specific controlled vocabulary and free-text terms related to traumatic brain injury, the cGAS-STING pathway, pharmacological inhibition and animal studies. Controlled vocabulary included terms related to traumatic brain injury and, where appropriate, indexing terms related to the cGAS-STING signaling pathway. Because indexing may be incomplete or delayed, particularly for recently introduced concepts, free-text terms were also incorporated, including terms related to traumatic brain injury (e.g., TBI, brain injury, head injury), cGAS, STING, STING1, TMEM173 and cGAS-STING pathway terminology, as well as terms related to pharmacological inhibition.

The search terms were combined using appropriate Boolean operators (AND, OR). Validated animal-study filters were incorporated into the PubMed and Embase search strategies to identify preclinical in vivo studies (S3 File) [26–28]. The complete electronic search strategies for MEDLINE, Embase and Web of Science are provided in S4 File.

The search strategies were peer reviewed by a second medical librarian using the Peer Review of Electronic Search Strategies (PRESS) validation [29]. All records were exported to Zotero^®^ for reference management and duplicate removal. Deduplicated records were then imported into Rayyan^®^ to support study selection.

### Study selection

Titles and abstracts were prescreened to determine study eligibility by two independent reviewers. Given that outcome measures are often unreported in animal study abstracts, full-text articles were systematically retrieved for further selection when abstracts information is insufficient. Full-text articles of potentially eligible studies were retrieved and independently assessed for inclusion by two reviewers. Any discrepancies in study inclusion during the two phases (title and abstract screening phase and full-text screening phase) were resolved through discussion and, if necessary, consultation with a third independent reviewer. Potentially eligible non-English full-text articles were considered eligible and, when required, translated using validated translation tools to determine eligibility.

### Inclusion criteria

Studies in any language were included if they:

(1) are preclinical in vivo studies published as full-text articles in peer-reviewed journals.
(2) use animal models of traumatic brain injury regardless of injury severity
(3) investigate pharmacological inhibition of the cGAS-STING pathway: eligible interventions will be classified a priori as either direct or indirect pharmacological inhibition of the cGAS-STING pathway. Direct inhibitors will be defined as pharmacological compounds whose primary or stated molecular target is cGAS or STING, including cGAS inhibitors, STING antagonists, or compounds directly blocking STING activation or STING-dependent signaling. Indirect inhibitors will be defined as pharmacological compounds that do not primarily target cGAS or STING but are reported to inhibit cGAS-STING pathway signaling through upstream, downstream or interacting mechanisms. Indirect inhibitors will be eligible only if the study provides experimental evidence linking the intervention to reduced cGAS-STING pathway activity, such as reduced cGAS or STING expression, reduced TBK1 or IRF3 activation, reduced type I interferon signaling. Broad anti-inflammatory or neuroprotective compounds without explicit assessment of cGAS-STING pathway involvement will not be considered eligible.
(4) comparator group receiving vehicle or no treatment with randomized or non-randomized design
(5) reporting at least one relevant outcome related to neuroinflammation (microglial or astrocytic reactivity), neurological function, or structural brain injury

The intervention group will include animals with a TBI from studies that evaluated the efficacy and/or the safety of a cGAS-STING pathway inhibitors in any dosing or frequency. Safety will be described when reported, including mortality, adverse events, body-weight changes and clinical signs of toxicity. The treatment should have been given after the induction of the TBI. The comparator group should be TBI animals who received only the vehicle or no treatment.

### Exclusion criteria

During the title and abstract screening phase, studies were excluded if they:

(1) do not involve an *in vivo* animal model of traumatic brain injury
(2) are not related to the inhibition of the cGAS-STING pathway
(3) reviews, editorials, commentaries, or conference abstracts

During the full-text screening phase, studies were excluded if they:

(1) are only *in vitro* or *ex vivo* studies
(2) do not investigate pharmacological inhibition of the cGAS-STING pathway: genetic knockout models without pharmacological intervention

Studies were excluded from the quantitative synthesis if relevant outcome data are not reported or if effect size estimates cannot be obtained after attempts at numerical extraction from figures or conversion of reported measures. Such studies were retained and included in the qualitative synthesis when appropriate.

### Data extraction

After deduplication, data will be independently extracted from the text and graphs of each paper by two independent investigators. We will use the online platform SyRF^®^ for data extraction. Any discrepancies will be resolved through discussion and, if necessary, consultation with a third independent reviewer. The corresponding authors of the studies included will be contacted via email to request any missing data. In the absence of a response, a single follow-up email will be sent after 15 days. When only graphical presentation will be available, values for mean and standard deviation (SD) or standard error of the mean (SEM) will be obtained using WebPlotDigitizer^®^ under high magnification.

Data collection items are presented in Table 2.

**Table 2:** Data collection items.

Domains	Variables
Study characteristics	DOI, year of publication, name of journal, country where the study is conducted (in case of collaboration: country of the leader), funding source, experimental design: randomized or not, stated objectives as reported in the manuscript, ARRIVE guideline followed, registered and in which register
Animals	Species, strain, sex, age, weight, total number of animals included, number per group, housing conditions
Models	Weight drop, controlled cortical impact, fluid percussion, other Injury severity: mild, moderate or severe Injury parameters: impact depth, pressure, velocity Injury location: cortical, diffuse, other Anesthesia used: agent, dose and duration when reported Mortality of the model
Intervention	Type of inhibitor, compound name, molecular target, dose, route of administration, timing post TBI, frequency, treatment duration
Control	Vehicle or no treatment Vehicle composition
Outcomes	Marker used (e.g., Iba1, GFAP, TNF-α, IL-1β, IL-6), brain region, methods, measurement type, unit, time post injury, mean, SD or SEM
Risk of bias	Baseline data, sequence generation, allocation concealment, random housing, blinding of investigators, random outcome assessment, blinding of the assessor, incomplete outcome data, selective outcome reporting and other sources of bias, blinded assessment of outcome
Quality	Statement of temperature control, appropriate animal model, sample size calculation, compliance with animal welfare regulations, statement of potential conflict of interests

ARRIVE: Animal Research: Reporting of In Vivo Experiments; DOI: digital object identifier; SD: standard deviation; SEM: standard error of the mean; TBI: traumatic brain injury.

### Outcomes

#### Primary outcome.

Microglial activation. The primary outcome will be the magnitude of microglial activation in brain tissue following traumatic brain injury, comparing pharmacological inhibition of the cGAS-STING pathway with vehicle or no intervention. Microglial activation will be assessed using quantitative measures of ionized calcium-binding adapter molecule 1 (Iba1)-positive cells (cell density, percentage area or fluorescence intensity) and/or markers of activated microglia, as reported in the included studies. When multiple microglial activation measures are available within the same study and at the same time point, Iba1-based quantitative outcomes (cell density or percentage area) will be prioritized for data extraction and pooling. If Iba1-based measures are unavailable, other validated markers of microglial activation will be used. To minimize selective outcome reporting, a pre-specified time window will be applied. For each study, the primary microglial outcome will be extracted from the first 72 hours post-injury time point. If no data are available within the pre-specified 72-hour window, the study will not contribute to the primary microglial/macrophage outcome analysis. Later time points will be extracted and reported separately as exploratory secondary analyses. When at least two independent studies report comparable later time points, these data will be pooled separately from the primary 72 hour analysis.

Microglial activation is selected as the primary outcome of this meta-analysis because it represents a central and early biological process in the pathophysiology of secondary brain injury following TBI. Microglia are the resident innate immune cells of the central nervous system and play a pivotal role in initiating, amplifying and sustaining neuroinflammatory responses after trauma [30]. Experimental models consistently demonstrate rapid microglial activation within hours following TBI. Pharmacological inhibition of the cGAS-STING pathway is expected to exert its primary biological effects at the level of innate immune activation, particularly within microglial populations. However, because Iba1 is expressed by both resident microglia and infiltrating macrophages, Iba1-based outcomes will be interpreted as markers of microglial and macrophage response. Similarly, increased Iba1-positive cell density, percentage area or fluorescence intensity does not necessarily indicate that these cells are activated. Therefore, when studies report additional information on microglial activation, such as morphological changes or additional markers such as CD68, TMEM 119, P2RY12 or co-localized markers, these data will be extracted and taken into account in the analysis and interpretation of Iba1-based outcomes. When sufficient data are available, sensitivity or subgroup analyses will be performed according to the type of quantification used, including cell density or count, percentage-stained area, fluorescence intensity, morphology-based measures and activation or phenotypic markers.

#### Secondary outcomes.

Astrocytic reactivity. Reactive astrogliosis, commonly assessed by glial fibrillary acidic protein (GFAP) expression, is another prominent feature of the post-traumatic inflammatory response and may contribute to blood-brain barrier dysfunction, tissue remodeling, and glial scar formation.

Astrocytic reactivity will be assessed using quantitative GFAP-based measures in brain tissue following TBI.

Eligible measurements will include GFAP-positive cell density, percentage area, fluorescence intensity, optical density, or equivalent tissue-based quantification methods reported in the included studies. When multiple GFAP-based measures are available within the same study and at the same time point, immunohistochemistry or immunofluorescence-based quantification will be prioritized for data extraction and pooling.

GFAP-based astrocytic reactivity will be extracted from the first 72 hours post-injury time window. Later GFAP time points will be extracted, documented, and analyzed separately when sufficient comparable data are available.

Neuroinflammatory cytokine expression. Change in pro-inflammatory cytokine levels (TNF-α, IL-1β, and IL-6) in brain tissue following TBI.

Eligible measurements will include both transcriptional and protein-level quantifications. Transcriptional measures will include quantitative polymerase chain reaction (PCR) or equivalent methods reporting fold-change, ΔΔCt-derived expression, or expression normalized to housekeeping genes. Protein-level measures will include enzyme-linked immunosorbent assays (ELISA), multiplex immunoassays (Luminex) or Western blot quantification.

Given potential differences between transcriptional and protein-level regulation, mRNA and protein outcomes will be analyzed separately when sufficient data are available. When pooling across methodologies is deemed appropriate, standardized mean differences (Hedges’ g) will be used to account for differences in measurement scales.

Cytokine expression will be extracted from the first 72 hours post-injury time window. Later cytokine time points will be extracted, documented and analyzed separately when sufficient comparable data are available.

When both mRNA and protein data are reported within the same study, these will be treated as distinct outcome measures within separate analyses to avoid unit-of-analysis errors.

Structural brain injury. Structural brain injury will be assessed using quantitative measures of tissue damage, including:

- Lesion volume or tissue loss measured by histology (cresyl violet or Nissl staining) or neuroimaging (magnetic resonance imaging or MRI).

When multiple structural outcomes are reported, absolute lesion volume (mm³) will be prioritized over relative measures. The primary time window for lesion volume will be the first 7 days post-injury.

Neurological functional outcomes. Functional recovery will include both motor and behavioral outcomes assessed after TBI.

Eligible measures will be:

- Motor outcomes: rotarod, beam walk/beam balance, grip strength, neurological severity score, open field locomotion metrics, gait analysis.
- Behavioral outcomes: morris water maze, novel object recognition, Y-maze or T-maze.

The primary functional outcome window will be 14–28 days post-injury. Functional outcomes assessed beyond 28 days will be extracted and analyzed separately as longer-term functional outcomes when sufficient comparable data are available. Motor and cognitive outcomes will be pooled separately.

Effect sizes will be oriented so that negative standardized mean difference (SMD) indicates benefit (reduced deficits / improved performance). For outcomes where higher values indicate worse performance (e.g., latency measures depending on test), directions will be harmonized a priori.

For each outcome domain, when multiple brain regions, time points, or assessment methods are reported within a single study, a single outcome per domain will be selected according to the pre-specified hierarchy and time windows to avoid selective reporting and unit-of-analysis errors.

When outcomes are reported using different scales or methodologies, effect sizes will be pooled using standardized mean differences (Hedges’ g), with directionality harmonized such that negative values indicate reduced neuroinflammation, reduced lesion size, or improved neurological function, as appropriate

Given variability in animal species, TBI models, inhibitors, and outcome time points, we anticipate moderate to substantial between study heterogeneity. Therefore, random-effects models will be used, and pooling will be restricted to outcomes reported by at least two independent studies within pre-specified time windows.

### Risk of bias assessment

To evaluate the quality of the studies and risk of any bias, two independent investigators will use the Systematic Review Center for Laboratory animal Experimentation (SYRCLE)’s risk of bias tool, which includes 10 defined criteria: (1) sequence generation, (2) baseline characteristics, (3) allocation concealment, (4) random housing, (5) blinding against performance bias, (6) random outcome assessment, (7) blinding against detection bias, (8) incomplete outcome data, (9) selective outcome reporting, and (10) other sources of biases including contamination and inappropriate influence of funders [31].

To assign a judgment of low, high, or unclear risk of bias to each item mentioned in the tool, the signaling questions will be used as indicated in the original article.

A “yes” judgment will indicate a low risk of bias, a “no” judgment will indicate high risk of bias, and if insufficient details were reported to assess the risk of bias properly, the judgment of bias will be recorded as “unclear”. Assessments will be done by at least two independent reviewers, and disagreements will be first resolved through consensus discussion and if necessary, by consulting a third reviewer. SYRCLE judgments will be made separately for each domain and will not be combined into a numerical summary score. Where required for subgroup or sensitivity analyses, studies will be categorized using an overall risk of bias judgement based on pre-specified key domains.

### Methodological and reporting quality

Methodological quality and translational relevance will be assessed using modified 9-item CAMARADES score, including peer reviewed publication, control of temperature, randomization, blinding during assessment of outcome, allocation concealment, appropriate animal model, sample size calculation, compliance with animal welfare regulations and declaration of conflicts of interest [32]. Anaesthetic regimen will not be included in the CAMARADES numerical score because the potential neuroprotective or anti-inflammatory properties of anaesthetic agents depend on the agent or combination of agents, dose, duration, timing and experimental context and cannot be reliably reduced to a binary quality item across studies [33]. Different studies may use different agents or combinations of agents, with variable doses, durations and timing of administration. Instead, anaesthetic agent, dose and duration will be extracted and reported separately as descriptive variables. These items will be reported descriptively and combined into a score (0–9). A score from 0 to 5 is considered as “low quality” and a score from 6 to 9 is considered as “high quality”. Assessments will be done by at least two independent reviewers, and disagreements will be first resolved through consensus discussion and if necessary, by consulting a third reviewer. This methodological quality score will be considered separately from the SYRCLE risk of bias assessment and will not be interpreted as a risk of bias score.

### Statistical analyses

First, we will summarize all the studies included in a narrative synthesis [34]. Then, we will perform quantitative synthesis when at least two independent studies report the same outcome.

If meta-analysis is not feasible, findings will be only summarized using a structured narrative synthesis. Included studies will be described according to animal species, TBI model, intervention characteristics, comparator, timing of treatment, outcome measures, and methodological features. Results will be grouped by outcome domain, including microglial activation, astrocytic reactivity, neuroinflammatory cytokine expression, lesion volume and neurological functional recovery. For each domain, we will summarize the direction, consistency, and magnitude of reported effects, together with the timing of assessment and the methods used for outcome quantification. Reasons why meta-analysis will not be considered appropriate, such as insufficient numbers of studies, incompatible outcome measures or incomplete reporting of numerical data, will be explicitly reported.

#### Effect measures.

For continuous outcomes measured on the same scale (e.g., lesion volume in mm³), we will use the mean difference with 95% confidence intervals (CIs). When outcomes are measured using different scales (e.g., motor and cognitive tests across platforms), we will use the standardized mean difference (SMD; Hedges g) with 95% CIs. For all outcomes, effect estimates will be oriented so that negative values indicate reduced neuroinflammation or lesion size or improved functional outcomes (as applicable); directions will be harmonized across studies prior to pooling.

#### Data handling and extraction.

When studies report variability as SEM, we will convert to SD using SD = SEM × square root of n. When multiple experimental arms share a single control group, we will avoid double-counting by either (i) combining relevant intervention arms when appropriate, or (ii) splitting the control group proportionally to avoid unit-of-analysis errors across comparisons according to Cochrane guidance for continuous outcomes [35]. When multiple publications report data from the same experimental cohort or overlapping animal experiments, these reports will be collated and treated as a single study to avoid double-counting. The most complete report will be used as the primary source for study characteristics and outcome data and additional reports will be used only to complement missing methodological or outcome information.

#### Unit of analysis and handling of dependence.

The primary unit of analysis will be the independent experiment. When a single study reports multiple time points for the same outcome, we will pre-specify a primary time point per domain to ensure comparability and avoid selective reporting:

- Microglial activation, astrocytic reactivity and cytokines quantification: primary time window the first 72 hours post-injury.
- Lesion volume: primary time point the first 7 days post-injury.
- Functional outcomes: primary time point between 14 and 28 days post-injury with separate pooling for motor and cognitive domains when feasible.

Studies reporting only later time points will be analyzed separately as later time-point outcomes when sufficient data are available.

To avoid unit-of-analysis errors, we will not include multiple dependent effect sizes from the same independent experiment in the same meta-analysis as if they were independent. When several eligible effect sizes are reported for the same outcome domain and time window within a single experiment, we will first apply the prespecified hierarchy for time point and outcome measure. For brain region, the injured or ipsilateral perilesional region will be prioritized. Cytokines will be analyzed separately by cytokine and by biological level when possible, distinguishing mRNA and protein outcomes. Multiple intervention dose arms sharing a common control group will be handled by combining relevant intervention arms when biologically appropriate or by splitting the shared control group to avoid double-counting. Sensitivity analyses using alternative outcome selections may be performed when outcome selection could influence the conclusions.

#### Meta-analysis model and heterogeneity.

Given the expected methodological diversity across animal species, traumatic brain injury models, and dosing regimens, a random-effects model will be used as the primary analytical approach. Between-study variance will be estimated using restricted maximum likelihood. For each pooled estimate, 95% confidence intervals will be calculated using the Hartung-Knapp adjustment when at least three independent studies are available. Conventional random-effects confidence intervals will be reported as sensitivity analyses when appropriate. Statistical heterogeneity will be assessed using τ², the I² statistic and Cochran’s Q test. A I² statistic values ≥75% will be considered indicative of substantial heterogeneity, according to conventional thresholds [36]. Prediction intervals will be reported when feasible, but τ², I² and prediction intervals will be interpreted cautiously when the number of included studies is small, particularly when fewer than 10 independent studies contribute to an analysis. Meta-regression will be performed when at least 10 independent studies or experiments are available per covariate.

#### Subgroup analyses.

Where sufficient data are available, defined as at least two independent studies per subgroup, we will conduct subgroup analyses by:

- Animal species (mouse *versus* rat)
- Sex (male versus female versus mixed)
- Age (adults versus immature brain)
- TBI model (controlled cortical impact, fluid percussion injury or weight drop)
- Timing of treatment (early < 24h versus delayed post-injury > 24h)
- Outcome assessment method (Iba1 immunohistochemistry versus other quantification approaches, when relevant)
- Intervention category defined as direct versus indirect pharmacological inhibition of the cGAS-STING pathway.
- Compound
- Route of administration
- Risk of bias (overall risk of bias judgement based on pre-specified key SYRCLE domains (lower risk versus higher risk))
- Methodological quality category based on the CAMARADES score (0–5 versus 6–9).

#### Sensitivity analyses.

Where sufficient data are available, we will perform sensitivity analyses to assess robustness by:

- Excluding studies judged at high risk of bias in one or more pre-specified key domains
- Using alternative random-effects estimators where appropriate for small numbers of studies
- Evaluating the influence of individual studies using leave-one-out analyses

#### Assessment of publication bias and small-study effects.

Publication bias and small-study effects will be evaluated when at least 10 studies are available for a given outcome, as recommended for reliable interpretation of funnel plot asymmetry [37]. Visual inspection of funnel plots (effect size versus standard error) will be performed to assess potential asymmetry. To statistically evaluate small-study effects, Egger’s regression test will be conducted when appropriate [38]. When at least 10 independent studies are available, small-study effects will primarily be assessed using a sample-size-based approach. Indeed, because Egger’s regression test may be biased when SMD are used, particularly in small preclinical animal study meta-analyses, it will not be used as the primary test for small-study effects. Egger’s test may be reported only as an exploratory sensitivity analysis and will be interpreted cautiously.

When evidence of asymmetry is detected, the trim-and-fill method will be applied to estimate the potential impact of unpublished or missing studies on the pooled effect size. Both the original and adjusted pooled estimates will be reported.

Given the known risk of selective reporting and publication bias in preclinical research, findings related to publication bias will be interpreted cautiously and discussed in the context of overall study quality and risk of bias assessments.

Analyses will be conducted using R (R Foundation for Statistical Computing), using established meta-analysis packages (meta). A two-sided p-value <0.05 will be considered statistically significant.

### Assessment of certainty of evidence

The certainty of evidence for all the outcomes will be assessed using the Grading of Recommendations Assessment, Development and Evaluation (GRADE) approach, adapted for preclinical animal studies [39]. Although GRADE was originally developed for clinical research, it is increasingly used in preclinical systematic reviews to evaluate the robustness and translational relevance of therapeutic effects observed in animal models.

For this review, GRADE will be applied to assess the certainty of evidence supporting the efficacy of pharmacological cGAS-STING pathway inhibition on neuroinflammation following TBI.

The certainty of evidence will be evaluated across the following GRADE domains: risk of bias, inconsistency, indirectness, imprecision, and publication bias. Risk of bias judgments will be informed by the SYRCLE risk of bias tool. Inconsistency will be assessed based on between-study heterogeneity and qualitative assessment of effect direction. Indirectness will be evaluated by examining discrepancies between the experimental animal models and the human disease, including species differences, TBI models, and the clinical relevance of selected outcome measures. These factors will be considered when interpreting the magnitude and applicability of the pooled effects. Imprecision will be evaluated based on confidence interval width and total sample size. Publication bias will be assessed when sufficient studies will be available.

The certainty of evidence for each outcome will be rated as high, moderate, low, or very low. Given the preclinical nature of the evidence, ratings will be interpreted cautiously and will be used to contextualize the strength and translational potential of therapeutic effects rather than to formulate clinical recommendations.

### Protocol amendments

Any protocol amendment made after registration will be documented and reported in the completed systematic review and meta-analysis. Amendments will also be updated in the PROSPERO record.

## Discussion

This protocol presents the framework for a prospectively planned systematic review and meta-analysis which will evaluate the efficacy of the pharmacological inhibition of the cGAS-STING pathway in animal models of TBI. Although growing experimental data suggest that the cGAS-STING axis plays a central role in post-traumatic neuroinflammation, the magnitude, consistency, and translational relevance of these findings have not yet been systematically evaluated.

Preclinical neurotrauma research is characterized by substantial heterogeneity in animal species, injury, treatment timing, outcome measures, and methodological quality. Such variability complicates interpretation of individual studies and may contribute to overestimation or underestimation of therapeutic effects. By quantitatively synthesizing available evidence and assessing risk of bias, this review will provide a more precise estimate of the impact of the pharmacological cGAS-STING pathway inhibition on microglial activation, astrocytic reactivity, cytokine expression, lesion volume and functional outcomes.

The translational failure of many neuroprotective therapeutic interventions in TBI underscores the importance of appraising preclinical evidence before progression to clinical investigation. If consistent and biologically meaningful effects are observed across independent models and species, this would strengthen the rationale for clinical investigation of cGAS-STING inhibitors. Conversely, if effects appear inconsistent, highly heterogeneous, or associated with substantial methodological limitations, this may indicate that additional preclinical optimization or improved experimental design are required before translation [40].

This review will also contribute to improving research practice in experimental neurotrauma. By systematically evaluating methodological quality using SYRCLE and CAMARADES tools, and by applying GRADE adapted for animal studies, we aim to identify design features associated with reduced risk of bias and enhanced translational relevance. Such information may inform future study design, including appropriate randomization, blinding, timing of intervention and outcome selection.

Several limitations inherent to preclinical systematic reviews should be acknowledged. First, the review will primarily rely on data available in the published literature [41]. It is well recognized that published studies are more likely to report statistically significant or positive findings, whereas neutral or negative results may remain unpublished or be disseminated only in grey literature sources such as theses, dissertations or conference proceedings [42]. This selective dissemination may contribute to publication bias and potentially lead to overestimation of treatment effects. Animal studies are also frequently underpowered and susceptible to selective outcome reporting, further increasing the risk of biased effect estimates. The decision to evaluate only peer-reviewed article is based on the need for complete methodological descriptions and sufficiently detailed numerical data required for risk of bias assessment and effect size calculation. Grey literature sources often lack standardized reporting of experimental design, variance measures, or sample sizes, which limits their suitability for rigorous quantitative synthesis.

Second, inbred strains commonly used in experimental research exhibit reduced genetic diversity and may display strain specific immune response profiles, whereas outbred strains present greater genetic variability [43–45]. Given that this review focuses on cGAS-STING inhibition, differences in genetic background and baseline immune reactivity across strains may influence the magnitude and direction of observed effects. Such variability may complicate interpretation and limit direct comparability across studies. Although we will attempt to mitigate these issues through predefined outcome hierarchies, time windows, subgroup analyses and sensitivity analyses, residual heterogeneity and reporting limitations are expected and should be recognized.

Third, TBI in humans is intrinsically heterogeneous. In clinical practice, TBI encompasses a wide spectrum of lesion types, including subdural and epidural hematomas, traumatic subarachnoid hemorrhage, cerebral contusions, diffuse axonal injury, petechial hemorrhages, and intraventricular hemorrhage, which frequently coexist within the same patient. As a result, each case of TBI represents a unique combination of injury patterns and systemic responses. In contrast, experimental models rely on standardized and highly controlled injury that intentionally isolate specific components of the injury process [46,47]. Although such reductionist approaches are essential for mechanistic exploration, they inevitably capture only selected dimensions of the human condition. This gap between clinical complexity and experimental simplification should be considered when interpreting the translational relevance of pooled preclinical findings in TBI.

To our knowledge, this will be the first systematic review to rigorously evaluate the risk of bias and methodological quality of studies investigating pharmacological cGAS-STING inhibition in experimental *in vivo* TBI models. This approach will help clarify whether the existing evidence is sufficiently robust to justify further potential progression toward clinical investigation.

## Supporting information

S1 File PRISMA-P Checklist. Completed PRISMA-P checklist for the systematic review and meta-analysis protocol.
(DOCX)

S2 File SYRCLE Checklist. Checklist used to support the preparation of the animal intervention systematic review protocol.
(DOCX)

S3 File Animal filter. Animal-study filters used to identify preclinical in vivo studies in the electronic searches.
(DOCX)

S4 File Search strategy. Complete database-specific search strategies for MEDLINE, Embase and Web of Science.
(DOCX)

## Abbreviations

ARRIVE: Animal Research: Reporting of In Vivo Experiments
CAMARADES: Collaborative Approach to Meta-Analysis and Review of Animal Data from Experimental Studies
CI: confidence interval
cGAS: cyclic GMP-AMP synthase
cGAS-STING: cyclic GMP-AMP synthase-stimulator of interferon genes
DNA: deoxyribonucleic acid
DOI: digital object identifier
ELISA: enzyme-linked immunosorbent assay
GFAP: glial fibrillary acidic protein
GRADE: Grading of Recommendations Assessment, Development and Evaluation
I²: I-squared statistic
Iba1: ionized calcium-binding adapter molecule 1
IL-1β: interleukin-1 beta
IL-6: interleukin-6
IRF3: interferon regulatory factor 3
mRNA: messenger ribonucleic acid
MRI: magnetic resonance imaging
NF-κB: nuclear factor kappa B
PICO: Population, Intervention, Comparator, Outcome
PCR: polymerase chain reaction
PRESS: Peer Review of Electronic Search Strategies
PRISMA: Preferred Reporting Items for Systematic Reviews and Meta-Analyses
PRISMA-P: Preferred Reporting Items for Systematic Review and Meta-Analysis Protocols
PROSPERO: International Prospective Register of Systematic Reviews
SD: standard deviation
SEM: standard error of the mean
SMD: standardized mean difference
STING: stimulator of interferon genes
SYRCLE: Systematic Review Centre for Laboratory Animal Experimentation
TBI: traumatic brain injury
TNF-α: tumor necrosis factor alpha
