# Supplementary material for: Pharmacological cGAS-STING pathway inhibition on neuroinflammation in animal models of traumatic brain injury: A systematic review and meta-analysis protocol
Source: PLoS One. 2026 Nov 6;21(11):e0362397. doi: 10.1371/journal.pone.0362397 (PMC13742951; doi:10.1371/journal.pone.0362397)
Supplement: Supplementary file 1 [file pone.0362397.s001.docx]

**PRISMA-P 2015 Checklist**

# **This checklist has been adapted for use with systematic review protocol submissions to BioMed Central journals from Table 3 in Moher D et al**:**** Preferred reporting items for systematic review and meta-analysis protocols (PRISMA-P) 2015 statement. *Systematic Reviews* 2015 ****4****:1

# An Editorial from the Editors-in-Chief of *Systematic Reviews* details why this checklist was adapted - **Moher D, Stewart L & Shekelle P**:**** Implementing PRISMA-P: recommendations for prospective authors. *Systematic Reviews* 2016 ****5****:15

| **Section/topic** | **#** | **Checklist item** | **Information reported** | | **Line number(s)** |
| --- | --- | --- | --- | --- | --- |
|  |  |  | **Yes** | **No** |  |
| **ADMINISTRATIVE INFORMATION** | | | | | |
| **Title** | | | | | |
| Identification | 1a | Identify the report as a protocol of a systematic review | X |  | Page 1; lines 1-2 |
| Update | 1b | If the protocol is for an update of a previous systematic review, identify as such | NA |  | NA |
| **Registration** | 2 | If registered, provide the name of the registry (e.g., PROSPERO) and registration number in the Abstract | X |  | Page 2; line 46 |
| **Authors** | | | | | |
| Contact | 3a | Provide name, institutional affiliation, and e-mail address of all protocol authors; provide physical mailing address of corresponding author | X |  | Page 1; lines 5-15 |
| Contributions | 3b | Describe contributions of protocol authors and identify the guarantor of the review | X |  | Page 31; lines 669-672 |
| **Amendments** | 4 | If the protocol represents an amendment of a previously completed or published protocol, identify as such and list changes; otherwise, state plan for documenting important protocol amendments | X |  | Page 26-27; lines 547-550 |
| **Support** | | | | | |
| Sources | 5a | Indicate sources of financial or other support for the review | X |  | Page 31; lines 667-668 |
| Sponsor | 5b | Provide name for the review funder and/or sponsor | X |  | Page 31: no funding |
| Role of sponsor/funder | 5c | Describe roles of funder(s), sponsor(s), and/or institution(s), if any, in developing the protocol | NA |  | NA |
| **INTRODUCTION** | | | | | |
| **Rationale** | 6 | Describe the rationale for the review in the context of what is already known | X |  | Pages 3-5; lines 51-100 |
| **Objectives** | 7 | Provide an explicit statement of the question(s) the review will address with reference to participants, interventions, comparators, and outcomes (PICO) | X |  | Page 5; lines 102-113; Table 1, pages 6-7 |
| **METHODS** | | | | | |
| **Eligibility criteria** | 8 | Specify the study characteristics (e.g., PICO, study design, setting, time frame) and report characteristics (e.g., years considered, language, publication status) to be used as criteria for eligibility for the review | X |  | Table 1, pages 6-7; page 8; lines 149-160; pages 10-11; lines 195-234 |
| **Information sources** | 9 | Describe all intended information sources (e.g., electronic databases, contact with study authors, trial registers, or other grey literature sources) with planned dates of coverage | X |  | Page 8; lines 149-160; page 9; lines 176-179; pages 11-12; lines 237-244 |
| **Search strategy** | 10 | Present draft of search strategy to be used for at least one electronic database, including planned limits, such that it could be repeated | X |  | S3 File and S4 File; page 9; lines 171-174 |
| ***STUDY RECORDS*** | | | | | |
| Data management | 11a | Describe the mechanism(s) that will be used to manage records and data throughout the review | X |  | Page 9; lines 176-179; page 11; lines 237-238 |
| Selection process | 11b | State the process that will be used for selecting studies (e.g., two independent reviewers) through each phase of the review (i.e., screening, eligibility, and inclusion in meta-analysis) | X |  | Page 9; lines 183-191; page 11; lines 223-234 |
| Data collection process | 11c | Describe planned method of extracting data from reports (e.g., piloting forms, done independently, in duplicate), any processes for obtaining and confirming data from investigators | X |  | Pages 11-12; lines 237-244; Table 2, page 13 |
| **Data items** | 12 | List and define all variables for which data will be sought (e.g., PICO items, funding sources), any pre-planned data assumptions and simplifications | X |  | Table 2, page 13 |
| **Outcomes and prioritization** | 13 | List and define all outcomes for which data will be sought, including prioritization of main and additional outcomes, with rationale | X |  | Pages 14-18; lines 251-361 |
| **Risk of bias in individual studies** | 14 | Describe anticipated methods for assessing risk of bias of individual studies, including whether this will be done at the outcome or study level, or both; state how this information will be used in data synthesis | X |  | Page 19; lines 362-380 |
| ***DATA*** | | | | | |
| **Synthesis** | 15a | Describe criteria under which study data will be quantitatively synthesized | X |  | Page 18; lines 358-361; page 20; lines 401-404 |
|  | 15b | If data are appropriate for quantitative synthesis, describe planned summary measures, methods of handling data, and methods of combining data from studies, including any planned exploration of consistency (e.g., *I* ^2^, Kendall’s tau) | X |  | Pages 21-23; lines 417-473 |
|  | 15c | Describe any proposed additional analyses (e.g., sensitivity or subgroup analyses, meta-regression) | X |  | Pages 23-24; lines 471-491; page 24; lines 493-499 |
|  | 15d | If quantitative synthesis is not appropriate, describe the type of summary planned | X |  | Pages 20-21; lines 406-415 |
| **Meta-bias(es)** | 16 | Specify any planned assessment of meta-bias(es) (e.g., publication bias across studies, selective reporting within studies) | X |  | Page 19; lines 364-370; pages 24-25; lines 501-517 |
| **Confidence in cumulative evidence** | 17 | Describe how the strength of the body of evidence will be assessed (e.g., GRADE) | X |  | Pages 25-26; lines 522-545 |
