# Supplementary material for: Pharmacological cGAS-STING pathway inhibition on neuroinflammation in animal models of traumatic brain injury: A systematic review and meta-analysis protocol
Source: PLoS One. 2026 Nov 6;21(11):e0362397. doi: 10.1371/journal.pone.0362397 (PMC13742951; doi:10.1371/journal.pone.0362397)
Supplement: Supplementary file 2 [file pone.0362397.s002.docx]

|  **Systematic Review Protocol for Animal Intervention Studies**  **Format by SYRCLE (**[**www.syrcle.nl**](http://www.syrcle.nl)**)**  **Version 2.0 (December 2014)** | | | | |
| --- | --- | --- | --- | --- |
| **Item #** | **Section/Subsection/Item** | **Description** | | **Check for approval** |
|  | A. General | | | |
| 1. | Title of the review | Efficacy of pharmacological cGAS-STING pathway inhibition on neuroinflammation in animal models of traumatic brain injury: A systematic review and meta-analysis protocol | | Check |
| 2. | Authors (names, affiliations, contributions) | Myriam Lamamri (MD) ^1,2^, Stéphanie Sigaut (MD, PhD) ^1,2^, Hélène Cwerman-Thibault (PhD) ^2^, Valérie Faivre (PhD) ^2^, Juliette Van Steenwinckel (PhD) ^2^, Vincent Degos (MD, PhD)^2,3^, Pierre Gressens (MD, PhD) ^2^, Alice Jacquens (MD, PhD)^2,3^  ^1^ Département d'anesthésie réanimation, AP-HP, Hôpital Beaujon, DMU PARABOL, Clichy, France.  ^2^ Université Paris Cité, Inserm, NeuroDiderot, F-75019 Paris, France  ^3^ Département d'anesthésie réanimation, AP-HP, Hôpital La Pitié Salpétrière, DMU DREAM, Paris, France  ML conceived the study. ML drafted the manuscript. AJ, SS, PG and VD contributed to the development of the methodology. JV, VF, HC revised the manuscript. All authors read and approved the final manuscript. | | Check |
| 3. | Other contributors (names, affiliations, contributions) |  | |  |
| 4. | Contact person + e-mail address | Dr Lamamri Myriam; myriam.lamamri@yahoo.fr | | Check |
| 5. | Funding sources/sponsors | None | | Check |
| 6. | Conflicts of interest | None | | Check |
| 7. | Date and location of protocol registration | PROSPERO - April 12, 2026 | | Check |
| 8. | Registration number (if applicable) | PROSPERO CRD420261367166 | | Check |
| 9. | Stage of review at time of registration | At the time of registration, the review was at the protocol development stage. Preliminary article identification had begun, but the final database search and study selection had not yet started. | | Check |
|  | B. Objectives | | | |
|  | Background | | | |
| 10. | What is already known about this disease/model/intervention? Why is it important to do this review? | Traumatic brain injury (TBI) is a major cause of mortality and long-term neurological disability worldwide. Secondary injury mechanisms, particularly neuroinflammation, play a key role in determining neurological outcomes after the initial trauma. The cyclic GMP-AMP synthase stimulator of interferon genes (cGAS-STING) pathway has recently been identified as a mediator of innate immune activation following brain injury. Activation of this pathway promotes microglial activation and the release of pro-inflammatory cytokines, contributing to neuronal damage and impaired recovery. Pharmacological inhibition of the cGAS-STING signaling pathway has therefore emerged as a promising neuroprotective strategy in experimental TBI models. However, the available preclinical evidence has not yet been systematically synthesized. | | Check |
|  | Research question | | | |
| 11. | Specify the disease/health problem of interest | Traumatic brain injury | | Check |
| 12. | Specify the population/species studied | All animals (any species, any severity model) | | Check |
| 13. | Specify the intervention/exposure | Pharmacological inhibition of the cGAS-STING pathway administered after TBI induction | | Check |
| 14. | Specify the control population | Vehicle treated or untreated TBI animals | | Check |
| 15. | Specify the outcome measures | The primary outcome will be quantitative microglial activation within 72 hours following injury. Secondary outcomes will include astrocytic reactivity assessed by GFAP, neuroinflammatory cytokine mRNA and protein expression (TNF-α, IL-1β, IL-6), lesion volume and neurological functional recovery. | | Check |
| 16. | State your research question (based on items 11-15) | Based on the PICO framework, the research question is defined as follows:  Population: In vivo animal models of TBI, regardless of species, sexes, ages or injury severity.  Intervention: Pharmacological inhibition of the cGAS-STING pathway administered after TBI induction.  Comparator : Vehicle-treated or untreated TBI animals.  Primary outcome : Quantitative microglial activation assessed within the first 72 hours post-injury.  Secondary outcomes : Astrocytic reactivity assessed by glial fibrillary acidic protein (GFAP); pro-inflammatory cytokine expression (mRNA and protein levels of TNF-α, IL-1β, and IL-6); lesion volume and neurological functional recovery. | | Check |
|  | C. Methods | | | |
|  | Search and study identification | | | |
| 17. | Identify literature databases to search (*e.g.* Pubmed, Embase, Web of science) | X MEDLINE via PubMed X Web of Science  □SCOPUS X EMBASE  □Other, namely:  □Specific journal(s), namely: | | Check |
| 18. | Define electronic search strategies (*e.g.* use the [step by step search guide^15^](http://www.ncbi.nlm.nih.gov/pmc/articles/PMC3265183/pdf/LA-11-087.pdf) and animal search filters[^20,^](http://www.ncbi.nlm.nih.gov/pmc/articles/PMC3104815/pdf/LA-09-117.pdf) [^21^](http://lan.sagepub.com/content/48/1/88.full.pdf+html)) | When available, please add a supplementary file containing your search strategy: Supplementary File 3 and File 4 | | Check |
| 19. | Identify other sources for study identification | □Reference lists of included studies □Books  □Reference lists of relevant reviews  □Conference proceedings, namely:  □Contacting authors/ organisations, namely:  □Other, namely: | | Check |
| 20. | Define search strategy for these other sources | N/A | | Check |
|  | Study selection | | | |
| 21. | Define screening phases (*e.g.* pre-screening based on title/abstract, full text screening, both) | During the title and abstract screening phase, studies will be excluded if they:   - do not involve an *in vivo* animal model of traumatic brain injury - are not related to the cGAS-STING pathway - reviews, editorials, commentaries, or conference abstracts   During the full-text screening phase, studies will be excluded if they:   - only *in vitro* or *ex vivo* studies - do not investigate pharmacological inhibition of the cGAS-STING pathway : genetic knockout models without pharmacological intervention | | Check |
| 22. | Specify (a) the number of reviewers per screening phase and (b) how discrepancies will be resolved | Two reviewers and any discrepancies in study inclusion will be resolved through discussion and, if necessary, consultation with a third independent reviewer. | | Check |
|  | *Define all inclusion and exclusion criteria based on:* | | | |
| 23. | Type of study (design) | Inclusion criteria: preclinical in vivo studies  Exclusion criteria: do not involve an *in vivo* animal model of traumatic brain injury or reviews, editorials, commentaries, or conference abstracts | | Check |
| 24. | Type of animals/population (*e.g.* age, gender, disease model) | Inclusion criteria: use animals’ models of traumatic brain injury regardless of injury severity, all species, all gender and all age.  Exclusion criteria: Not TBI or human | | Check |
| 25. | Type of intervention (*e.g.* dosage, timing, frequency) | Inclusion criteria: investigate pharmacological inhibition of the cGAS-STING pathway Exclusion criteria: do not investigate pharmacological inhibition of the cGAS-STING pathway: genetic knockout models without pharmacological intervention | | Check |
| 26. | Outcome measures | Inclusion criteria: quantitative microglial activation and astrocytic reactivity within 72 hours following injury and neuroinflammatory cytokine expression (TNF-α, IL-1β, IL-6), lesion volume, and neurological functional recovery  Exclusion criteria: | | Check |
| 27. | Language restrictions | Inclusion criteria: all language  Exclusion criteria: none | | Check |
| 28. | Publication date restrictions | Inclusion criteria: at any time  Exclusion criteria: None | | Check |
| 29. | Other | Inclusion criteria:NA  Exclusion criteria:NA | | Check |
| 30. | Sort and prioritize your exclusion criteria per selection phase | During the title and abstract screening phase, studies will be excluded if they:   - do not involve an *in vivo* animal model of traumatic brain injury - are not related to the cGAS-STING pathway - reviews, editorials, commentaries, or conference abstracts   During the full-text screening phase, studies will be excluded if they:   - only *in vitro* or *ex vivo* studies - do not investigate pharmacological inhibition of the cGAS-STING pathway : genetic knockout models without pharmacological intervention | | Check |
|  | Study characteristics to be extracted (for assessment of external validity, reporting quality) | | | |
| 31. | Study ID (*e.g.* authors, year) | DOI, year of publication, name of journal | | Check |
| 32. | Study design characteristics (*e.g.* experimental groups, number of animals) | country where the study is conducted (in case of collaboration : country of the leader), funding source, experimental design : randomized or not, stated objectives as reported in the manuscript, ARRIVE guideline followed, registered and in which register | | Check |
| 33. | Animal model characteristics (*e.g.* species, gender, disease induction) | Species, strain, sex, age, weight, total number of animals included number per group, housing conditions  Weight drop, controlled cortical impact, fluid percussion, other  Injury severity : mild, moderate or severe  Injury parameters : impact depth, pressure, velocity  Injury location : cortical, diffuse, other  Anesthesia used : type and dose  Mortality of the model | | Check |
| 34. | Intervention characteristics (*e.g.* intervention, timing, duration) | Type of inhibitor, compound name, molecular target, dose, route of administration, timing post TBI, frequency, treatment duration | | Check |
| 35. | Outcome measures | Marker used, brain region, methods, measurement type, unit, time post injury, mean, SD or SEM | | Check |
| 36. | Other (*e.g.* drop-outs) | Baseline data, sequence generation, allocation concealment, random housing, blinding of investigators, random outcome assessment, blinding of the assessor, incomplete outcome data, selective outcome reporting and other source of bias, blinded assessment of outcome | | Check |
|  | Assessment risk of bias (internal validity) or study quality | | | |
| 37. | Specify (a) the number of reviewers assessing the risk of bias/study quality in each study and (b) how discrepancies will be resolved | Two independent investigators and disagreements will be first resolved through consensus discussion and if necessary, by consulting a third reviewer. | | Check |
| 38. | Define criteria to assess (a) the internal validity of included studies (*e.g.* selection, performance, detection and attrition bias) and/or (b) other study quality measures (*e.g.* reporting quality, power) | X By use of [SYRCLE's Risk of Bias tool^4^](http://www.biomedcentral.com/1471-2288/14/43/abstract)  □By use of SYRCLE’s Risk of Bias tool, adapted as follows:  X By use of [CAMARADES' study quality checklist, e.g ^22^](http://www.ncbi.nlm.nih.gov/pubmed/15060322)  □By use of CAMARADES' study quality checklist, adapted as follows:  □Other criteria, namely: | | Check |
|  | Collection of outcome data | | | |
| 39. | For each outcome measure, define the type of data to be extracted (*e.g.* continuous/dichotomous, unit of measurement) | Microglial activation will be assessed using quantitative measures of IBA1-positive cells (cell density, percentage area, or fluorescence intensity) and/or markers of activated microglia, as reported in the included studies. When multiple microglial activation measures are available within the same study and at the same time point, Iba1-based quantitative outcomes (cell density or percentage area) will be prioritized for data extraction and pooling. If Iba1-based measures are unavailable, other validated markers of microglial activation will be used. For each study, the primary microglial outcome will be extracted from the 72 first hours post-injury time point, using the value closest to 48 hours. If no data are available within this window, the closest reported time point will be used and documented.  Reactive astrogliosis, commonly assessed by glial fibrillary acidic protein (GFAP) expression, is another prominent feature of the post-traumatic inflammatory response and may contribute to blood-brain barrier dysfunction, tissue remodeling, and glial scar formation.  Astrocytic reactivity will be assessed using quantitative GFAP-based measures in brain tissue following traumatic brain injury.  Eligible measurements will include GFAP-positive cell density, percentage area, fluorescence intensity, optical density, or equivalent tissue-based quantification methods reported in the included studies. When multiple GFAP-based measures are available within the same study and at the same time point, immunohistochemistry or immunofluorescence-based quantification will be prioritized for data extraction and pooling.  GFAP-based astrocytic reactivity will be extracted from the first 72 hours post-injury time window, prioritizing the value closest to 48 hours. If no data are available within this window, the closest reported time point will be used and documented.  Change in pro-inflammatory cytokine levels (TNF-α, IL-1β, and IL-6) in brain tissue following TBI.  Eligible measurements will include both transcriptional and protein-level quantifications. Transcriptional measures will include quantitative PCR or equivalent methods reporting fold-change, ΔΔCt-derived expression, or expression normalized to housekeeping genes. Protein-level measures will include enzyme-linked immunosorbent assays (ELISA), multiplex immunoassays (Luminex) or Western blot quantification.  Structural brain injury will be assessed using quantitative measures of tissue damage, including:  -Lesion volume or tissue loss measured by histology (cresyl violet or Nissl staining) or neuroimaging (MRI).  When multiple structural outcomes are reported, absolute lesion volume (mm³) will be prioritized over relative measures. The primary time window for lesion volume will be the first 7 days post-injury. | | Check |
| 40. | Methods for data extraction/retrieval (*e.g.* first extraction from graphs using a digital screen ruler, then contacting authors) | The corresponding authors of the studies included will be contacted via email to request any missing data. In the absence of a response, a single follow-up email will be sent after 15 days. When only graphical presentation will be available, values for mean and SD or SEM will be obtained using WebPlotDigitizer® under high magnification. | | Check |
| 41. | Specify (a) the number of reviewers extracting data and (b) how discrepancies will be resolved | After deduplication, data will be extracted from the text and graphs of each paper by two independent investigators. Any discrepancies will be resolved through discussion and, if necessary, consultation with a third independent reviewer. | | Check |
|  | Data analysis/synthesis | | | |
| 42. | Specify (per outcome measure) how you are planning to combine/compare the data (*e.g.* descriptive summary, meta-analysis) | Data will be synthesized descriptively and, where appropriate, quantitatively using random-effects meta-analysis. Effect sizes will be calculated using mean differences or standardized mean differences (Hedges’ g) with 95% confidence intervals. Analyses will be conducted separately for each outcome domain, with methodological stratification when necessary. In the presence of substantial heterogeneity or insufficient data, a narrative synthesis will be provided | | Check |
| 43. | Specify (per outcome measure) how it will be decided whether a meta-analysis will be performed | We will summarized all the studies included in a narrative synthesis. Then, we will perform quantitative synthesis when at least two independent studies report the same outcome. When meta-analysis is not feasible, findings will be only summarized narratively. | | Check |
|  | *If a meta-analysis seems feasible/sensible, specify (for each outcome measure):* | | | |
| 44. | The effect measure to be used (*e.g.* mean difference, standardized mean difference, risk ratio, odds ratio) | For continuous outcomes, effect sizes will be expressed as mean differences (MD) when outcomes are measured on the same scale, and as standardized mean differences (SMD; Hedges’ g) when different scales or measurement methods are used. All effect estimates will be reported with 95% confidence intervals | | Check |
| 45. | The statistical model of analysis (*e.g.* random or fixed effects model) | A random-effects model will be used for all meta-analyses to account for expected between-study heterogeneity | | Check |
| 46. | The statistical methods to assess heterogeneity (*e.g.* I^2^, Q) | Statistical heterogeneity will be assessed using the I² statistic, with values ≥75% considered indicative of substantial heterogeneity. The Chi-square (Q) test will also be used, with a p-value <0.10 indicating significant heterogeneity. | | Check |
| 47. | Which study characteristics will be examined as potential source of heterogeneity (subgroup analysis) | Where sufficient data are available, we will conduct subgroup analyses by:   - Animal species (mouse *versus* rat) - Sex (male versus female versus mixed) - TBI model (controlled cortical impact, fluid percussion injury or weight drop) - Timing of treatment (early versus delayed post-injury, based on study definitions) - Outcome assessment method (Iba1 immunohistochemistry versus other quantification approaches, when relevant) - Risk of bias (overall risk of bias judgement based on pre-specified key SYRCLE domains (lower risk versus higher risk)) - Methodological quality category based on the CAMARADES score (0-5 versus 6-10). | | Check |
| 48. | Any sensitivity analyses you propose to perform | - Excluding studies judged at high risk of bias in one or more pre-specified key domains - Using alternative random-effects estimators where appropriate for small numbers of studies - Evaluating the influence of individual studies using leave-one-out analyses | | Check |
| 49. | Other details meta-analysis (*e.g.* correction for multiple testing, correction for multiple use of control group) | To avoid unit-of-analysis errors, studies with multiple intervention arms sharing a common control group will be handled by combining intervention groups or splitting the control group according to Cochrane recommendations. When multiple outcomes within the same domain are reported, a single outcome will be selected based on predefined criteria. No formal correction for multiple testing will be applied, and results will be interpreted cautiously. | | Check |
| 50. | The method for assessment of publication bias | Publication bias and small-study effects will be evaluated when at least 10 studies are available for a given outcome, as recommended for reliable interpretation of funnel plot asymmetry. When evidence of asymmetry is detected, the trim-and-fill method will be applied to estimate the potential impact of unpublished or missing studies on the pooled effect size. | | Check |
|  | | | | |
| Final approval by (names, affiliations): Alice Jacquens (MD, PhD)^2,3^  ^2^ Université Paris Cité, Inserm, NeuroDiderot, F-75019 Paris, France  ^3^ Département d'anesthésie réanimation, AP-HP, Hôpital La Pitié Salpétrière, DMU DREAM, Paris, France | |  | Date: 07/09/2026 | |
