# Supplementary material for: Pharmacological cGAS-STING pathway inhibition on neuroinflammation in animal models of traumatic brain injury: A systematic review and meta-analysis protocol
Source: PLoS One. 2026 Nov 6;21(11):e0362397. doi: 10.1371/journal.pone.0362397 (PMC13742951; doi:10.1371/journal.pone.0362397)
Supplement: Supplementary file 4 [file pone.0362397.s004.docx]

**S4 File: Search concepts, controlled vocabulary, free-text terms and database-specific search yields.**

**A. Pubmed**

| **Block** | **Search concept** | **Executed search equation (PubMed)** | **Records, n** |
| --- | --- | --- | --- |
| **BLOCK 1: POPULATION (P) - traumatic brain injury** |  |  |  |
| 1 | TBI controlled vocabulary | "Brain Injuries, Traumatic"[Mesh] OR "Craniocerebral Trauma"[Mesh] OR "Brain Concussion"[Mesh] OR "Brain Contusion"[Mesh] OR "Head Injuries, Closed"[Mesh] | 193,892 |
| 2 | TBI text words | TBI[tiab] OR "traumatic brain injury"[tiab] OR "traumatic brain injuries"[tiab] OR "brain trauma"[tiab] OR "head trauma"[tiab] OR "traumatic head injury"[tiab] OR "traumatic head injuries"[tiab] OR "craniocerebral trauma"[tiab] OR "cranio-cerebral trauma"[tiab] OR "closed head injury"[tiab] OR "closed head injuries"[tiab] OR "brain concussion"[tiab] OR "brain contusion"[tiab] OR "cerebral contusion"[tiab] OR "controlled cortical impact"[tiab] OR "fluid percussion injury"[tiab] OR "fluid percussion injuries"[tiab] OR "weight drop"[tiab] | 85,054 |
| 3 | TBI block | #1 OR #2 | 225,786 |
| **BLOCK 2: TARGET / MECHANISM - cGAS-STING signaling** |  |  |  |
| 4 | cGAS-STING/STING controlled vocabulary | "cGAS-STING Signaling Pathway"[Mesh] OR "STING Protein"[Mesh] | 3,577 |
| 5 | cGAS-STING/STING text words | cGAS[tiab] OR "cyclic GMP-AMP synthase"[tiab] OR MB21D1[tiab] OR STING[tiab] OR STING1[tiab] OR TMEM173[tiab] OR "STING protein"[tiab] OR "stimulator of interferon genes"[tiab] OR "stimulator of interferon gene"[tiab] OR "stimulator of interferon response cGAMP interactor 1"[tiab] OR "transmembrane protein 173"[tiab] OR "cGAS-STING"[tiab] OR "cGAS STING"[tiab] OR "cGAS/STING"[tiab] | 15,620 |
| 6 | Pathway block | #4 OR #5 | 15,672 |
| **BLOCK 3: INTERVENTION - pharmacological inhibition** |  |  |  |
| 7 | Drug-effects / inhibitors controlled vocabulary | "cGAS-STING Signaling Pathway/drug effects"[Mesh] OR "STING Protein/antagonists and inhibitors"[Mesh] OR "STING Protein/drug effects"[Mesh] | 15 |
| 8 | Pharmacological inhibition and compound text words | inhibit*[tiab] OR antagonis*[tiab] OR block*[tiab] OR suppress*[tiab] OR pharmacolog*[tiab] OR drug*[tiab] OR "small molecule"[tiab] OR "small molecules"[tiab] OR "C-176"[tiab] OR C176[tiab] OR "C-178"[tiab] OR C178[tiab] OR "H-151"[tiab] OR H151[tiab] OR "RU.521"[tiab] OR "RU-521"[tiab] OR RU521[tiab] OR "SN-011"[tiab] OR SN011[tiab] OR G150[tiab] OR "PF-06928215"[tiab] | 6,526,633 |
| 9 | Pharmacological intervention block | #7 OR #8 | 6,526,635 |
| **BLOCK 4 : animal filters** | Animal filter | #10 | 5,472,584 |
| **BLOCK 5: FINAL COMBINATION** | Main intersection | #3 AND #6 AND #9 AND #10 | 44 |

**B. Embase**

| **Block** | **Concept** | **Search expression** | **Records, n** |
| --- | --- | --- | --- |
| **BLOCK 1: POPULATION (P) - traumatic brain injury** |  |  |  |
| #1 | Patient - controlled vocabulary | 'traumatic brain injury'/exp OR 'traumatic brain injury' OR 'brain injury'/exp OR 'brain injury' OR 'head injury'/exp OR 'head injury' OR 'brain contusion'/exp OR 'brain contusion' OR 'brain concussion'/exp OR 'brain concussion' | 457,589 |
| #2 | Patient - keywords | 'traumatic brain injury':ti,ab,kw OR 'traumatic brain injuries':ti,ab,kw OR tbi:ti,ab,kw OR 'brain injury':ti,ab,kw OR 'brain injuries':ti,ab,kw OR 'head injury':ti,ab,kw OR 'head injuries':ti,ab,kw OR 'traumatic head injury':ti,ab,kw OR 'traumatic head injuries':ti,ab,kw OR 'brain trauma':ti,ab,kw OR 'head trauma':ti,ab,kw OR 'craniocerebral trauma':ti,ab,kw OR 'cranio-cerebral trauma':ti,ab,kw OR 'closed head injury':ti,ab,kw OR 'closed head injuries':ti,ab,kw OR concussion:ti,ab,kw OR concussions:ti,ab,kw OR 'brain concussion':ti,ab,kw OR 'brain contusion':ti,ab,kw OR 'cerebral contusion':ti,ab,kw OR 'controlled cortical impact':ti,ab,kw OR 'fluid percussion injury':ti,ab,kw OR 'fluid percussion':ti,ab,kw OR 'weight drop':ti,ab,kw OR 'weight-drop':ti,ab,kw OR 'impact acceleration':ti,ab,kw OR 'blast-induced traumatic brain injury':ti,ab,kw OR 'blast traumatic brain injury':ti,ab,kw | 222,240 |
| #3 | Patient block | #1 OR #2 | 484,078 |
| **BLOCK 2: TARGET / MECHANISM - cGAS-STING signaling** |  |  |  |
| #4 | cGAS-STING/STING pathway keywords | cgas:ti,ab,kw OR 'c-gas':ti,ab,kw OR 'cyclic gmp-amp synthase':ti,ab,kw OR 'cyclic gmp amp synthase':ti,ab,kw OR 'cyclic guanosine monophosphate-adenosine monophosphate synthase':ti,ab,kw OR 'cgamp synthase':ti,ab,kw OR mb21d1:ti,ab,kw OR sting:ti,ab,kw OR sting1:ti,ab,kw OR tmem173:ti,ab,kw OR mita:ti,ab,kw OR mpys:ti,ab,kw OR eris:ti,ab,kw OR 'sting protein':ti,ab,kw OR 'stimulator of interferon genes':ti,ab,kw OR 'stimulator of interferon gene':ti,ab,kw OR 'transmembrane protein 173':ti,ab,kw OR 'cgas-sting':ti,ab,kw OR 'cgas sting':ti,ab,kw OR 'cgas/sting':ti,ab,kw | 22,162 |
| **BLOCK 3: INTERVENTION - pharmacological inhibition** |  |  |  |
| #5 | Pharmacological inhibition and compound keywords | inhibit*:ti,ab,kw OR inhibition:ti,ab,kw OR inhibitor*:ti,ab,kw OR antagonis*:ti,ab,kw OR antagoniz*:ti,ab,kw OR antagonist*:ti,ab,kw OR block*:ti,ab,kw OR blockade:ti,ab,kw OR suppress*:ti,ab,kw OR attenuat*:ti,ab,kw OR pharmacolog*:ti,ab,kw OR drug*:ti,ab,kw OR 'small molecule':ti,ab,kw OR 'small molecules':ti,ab,kw OR 'c-176':ti,ab,kw OR c176:ti,ab,kw OR 'c-178':ti,ab,kw OR c178:ti,ab,kw OR 'h-151':ti,ab,kw OR h151:ti,ab,kw OR 'ru.521':ti,ab,kw OR 'ru-521':ti,ab,kw OR ru521:ti,ab,kw OR 'sn-011':ti,ab,kw OR sn011:ti,ab,kw OR g150:ti,ab,kw OR 'pf-06928215':ti,ab,kw OR gsk2656157:ti,ab,kw OR 'gsk-2656157':ti,ab,kw OR 'gsk 2656157':ti,ab,kw OR tudca:ti,ab,kw OR 'tauroursodeoxycholic acid':ti,ab,kw | 9,031,318 |
| **BLOCK 4 : animal filters** |  | #6 | 6,523,871 |
| **BLOCK 5: FINAL COMBINATION** | Main intersection | #3 AND #4 AND #5 AND #6 | 109 |

**C. Web of science**

| **Line** | **Concept** | **Search expression** | **Records, n** |
| --- | --- | --- | --- |
| #1 | Population - traumatic brain injury | TS=("traumatic brain injury" OR "traumatic brain injuries" OR TBI OR "brain injury" OR "brain injuries" OR "head injury" OR "head injuries" OR "traumatic head injury" OR "traumatic head injuries" OR "brain trauma" OR "head trauma" OR "craniocerebral trauma" OR "cranio-cerebral trauma" OR "closed head injury" OR "closed head injuries" OR concussion* OR "brain concussion" OR "brain contusion" OR "brain contusions" OR "cerebral contusion" OR "cerebral contusions" OR "controlled cortical impact" OR "fluid percussion injury" OR "fluid percussion injuries" OR "fluid percussion" OR "weight drop" OR "weight-drop" OR "impact acceleration" OR "blast-induced traumatic brain injury" OR "blast traumatic brain injury") | 208225 |
| #2 | Target/mechanism - cGAS-STING signaling | TS=("cGAS" OR "CGAS" OR "c-GAS" OR "cyclic GMP-AMP synthase" OR "cyclic GMP AMP synthase" OR "cGAMP synthase" OR MB21D1 OR C6orf150 OR STING OR STING1 OR TMEM173 OR MITA OR MPYS OR ERIS OR "STING protein" OR "stimulator of interferon gene" OR "stimulator of interferon genes" OR "transmembrane protein 173" OR "cGAS-STING" OR "cGAS STING" OR "cGAS/STING" OR "cGAS-cGAMP-STING") | 28771 |
| #3 | Intervention - pharmacological inhibition | TS=(inhibit* OR inhibition OR inhibitor* OR antagonis* OR antagoniz* OR antagonist* OR block* OR blockade OR suppress* OR attenuat* OR pharmacolog* OR modulat* OR "small molecule" OR "small molecules" OR "C-176" OR C176 OR "C-178" OR C178 OR "H-151" OR H151 OR "RU.521" OR "RU-521" OR RU521 OR "SN-011" OR SN011 OR G150 OR "PF-06928215" OR GSK2656157 OR "GSK-2656157" OR "GSK 2656157" OR "Cl-amidine" OR "Cl amidine" OR chloramidine OR TUDCA OR "tauroursodeoxycholic acid" OR "tauroursodeoxycholate") | 9277439 |
| #4 | Final combination | #1 AND #2 AND #3 | 97 |

Validated animal filters were applied in PubMed and Embase. No validated animal filter was applied in Web of Science.
